# Effects of intranasal kinetic oscillation stimulation on heart rate variability

**DOI:** 10.1111/anec.12474

**Published:** 2017-06-07

**Authors:** Markus Jerling, Iwona Cygankiewicz, Nabil Al‐Tawil, Borje Darpo, Anders Ljungström, Wojciech Zareba

**Affiliations:** ^1^ Markus Jerling Consulting AB Bromma Sweden; ^2^ Department of Electrocardiology Medical University of Lodz Lodz Poland; ^3^ Karolinska Trial Alliance Phase I Unit Karolinska University Hospital Huddinge Sweden; ^4^ iCardiac Technologies, Inc. Rochester NY USA; ^5^ Division of Cardiovascular Medicine Department of Clinical Sciences Danderyd's Hospital Karolinska Institutet Stockholm Sweden; ^6^ ProgStat AB Stockholm Sweden; ^7^ Heart Research Follow Up Program University of Rochester Medical Center Rochester NY USA

**Keywords:** healthy subjects, heart rate variability, intranasal, kinetic oscillation stimulation

## Abstract

**Background:**

Kinetic oscillation stimulation in the nasal cavity (KOS) has been shown to have positive symptomatic effects in subjects with non‐allergic rhinitis and in patients with migraine.

**Methods:**

To evaluate the effect of KOS on autonomic function, we assessed heart rate variability (HRV) in this small exploratory study in 12 healthy subjects. KOS treatment was performed using a minimally invasive system with a single‐use catheter inserted into the nasal cavity. During treatment, the tip was inflated and oscillated with a mean pressure of 95 millibar and amplitude of the oscillations of 100 millibar at a frequency of 68 Hz. Treatment was given for 15 minutes sequentially on each side. Heart rate variability was assessed during five 30‐minutes periods before, during and immediately after KOS treatment and 3.5 hours thereafter. KOS resulted in a substantial reduction of HRV.

**Results:**

As compared to baseline recorded during 30 minutes preceding treatment, VLF was reduced by 65%, LF by 55%, the ratio LF/HF by 44%, with somewhat smaller observed effects in the time domain; SDNN and RMSDD were reduced by of 36% and 18%, respectively. Heart rate remained stable during treatment with minimal mean changes from 68 ± 7 bpm before to 68 ± 9 and 69 ± 9 bpm during and after treatment. Reduction of HRV parameters was consistently seen in all subjects, with rapid onset and return towards baseline values during post‐treatment observation periods.

**Conclusions:**

KOS has an effect on the autonomic balance with pronounced heart‐rate independent reduction on HRV.

## 1 INTRODUCTION

The Chordate System is a medical device that is used to administer kinetic oscillation stimulation (KOS) in the nasal cavity. The treatment is administered by inserting into either side of the nasal cavity a balloon, which is connected to an external air device exerting an oscillating pressure. The device is CE marked and permitted for usage within the EU for the treatment of nonallergic rhinitis (Juto & Axelsson, 2014). A small, randomized, placebo‐controlled pilot study has demonstrated positive effects on migraine with pronounced reduction in pain intensity during ongoing attacks (Juto & Hallin, 2015).

The mechanism of action of the KOS treatment is not fully elucidated and one hypothesis is that it alters vagal tone through a central mechanism. There have also been suggestions that patients with severe and disabling migraine have impaired autonomic function (Shechter, Stewart, Silberstein, & Lipton, 2002), which could suggest that KOS is effective due to altered autonomic tone.

The concept of heart rate variability (HRV) has for close to 50 years received extensive attention in different areas of medical research. A guideline on the standards of measurement, physiological interpretation, and clinical use was issued in 1996 by the Task Force of The European Society of Cardiology and The North American Society of Pacing and Electrophysiology, and the method has been successfully used to identify populations at high risk of cardiovascular events (Task Force of the European Society of Cardiology and the North American Society of Pacing and Electrophysiology, 1996; Sassi et al., 2015). The autonomic nervous system activity has direct effects on HRV parameters and, as an example, the high‐frequency (HF) component in the frequency domain is thought to predominantly reflect vagal tone. For other spectral domains, low frequency (LF), very low frequency (VLF), and ultralow frequency (ULF), the relationships are more complex and vagal and sympathetic activity, baroreflex function, and neuroendocrine factors may contribute. Consequently, HRV analyses have been widely applied to evaluate autonomic status both in healthy subjects and in patients with cardiovascular disease (Sassi et al., 2015).

To evaluate the effect of KOS on autonomic function, we incorporated an assessment of HRV and, on an exploratory basis, biomarkers of vagal tone in this small study in essentially healthy subjects, in addition to an evaluation of safety and tolerability of the treatment. Biomarkers/assessments included in the study were measurement of gastrin, which is considered a sensitive marker of vagal activity (Debas & Carvajal, 1994), cytokines (Borovikova et al., 2000), and spirometry, since bronchial tone is partly regulated by the parasympathetic system and increased vagal activity may elicit bronchoconstriction.

## 2 MATERIALS AND METHODS

### 2.1 Study design and subjects

**Figure 1:**
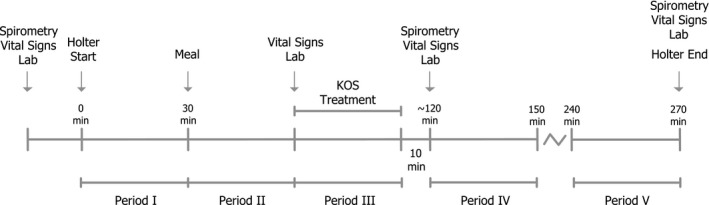
Schedule of events. Study assessments and periods for heart rate variability (HRV) analyses are shown in relation to meal and kinetic oscillation stimulation (KOS) treatment. Period 2 was immediately before KOS treatment, whereas period 3 was during treatment

This was an open‐label, single‐center safety study in male and female subjects who were treated with KOS with the chordate system on one occasion. Twelve healthy subjects were to be enrolled into the study. An outline of study events is given in Figure 1. At screening, vital signs and a standard 12‐lead ECG were recorded. Samples were taken for hematology, serum chemistries, HIV/hepatitis screening, and urinalyses and each subject underwent a physical examination. At the treatment visit, subjects arrived fasting in the morning and the following procedures were performed: control of continued eligibility criteria, measurement of vital signs and blood draws for laboratory variables, and for serum gastrin level and a panel of cytokines: TNF‐alpha and the interleukins 1b, 6, 8, and 10 and a lung function test with spirometry. A continuous ECG (Holter) recording was thereafter started. Subjects were instructed to avoid any strenuous exercise throughout the treatment visit.

Thirty minutes after initiation of the continuous ECG recording, a standardized meal was served, which was to be completed within 20 min. Thirty minutes after completion of the meal, vital signs assessment and blood draws for laboratory variables were performed. Immediately thereafter, KOS treatment was started with a total duration of approximately 30 min. To ensure comparable experimental conditions, subjects remained in a resting seated position during KOS treatment and all time windows during which HRV analysis was performed (periods I through V, as described below).

Ten minutes after end of treatment with KOS, vital signs assessment, blood draws for laboratory variables, including gastrin and cytokines, and spirometry were performed.

Three hours after end of KOS treatment, vital signs assessment, blood draws for laboratory variables, and spirometry were performed. The continuous ECG recording was thereafter terminated and subjects were discharged. At a follow‐up telephone call 5–7 days after the treatment visit, data for adverse events were collected.

The study was conducted in accordance with the relevant articles of the “Declaration of Helsinki” and International Conference on Harmonization Good Clinical Practice consolidated guidelines. All subjects gave their written consent to the study. The study commenced after authorization by the Medical Products Agency in Sweden and the Ethics Committee at the Karolinska Institute.

### 2.2 KOS treatment

Kinetic oscillation stimulation treatment was performed as previously described (Juto & Axelsson, 2014). During KOS treatment, subjects were comfortably resting in a chair. KOS treatment was administered using a minimally invasive system. It consisted of a controller and a single‐use catheter and a headband from Chordate Medical AB, Stockholm, Sweden. The controller was connected to the catheter. The headband was used to secure the position of the catheter. The catheter, with a coating of lubricating paraffin, was inserted into the nasal cavity, on either side. During KOS treatment, the tip was inflated and oscillated for 15 min with a mean pressure of 95 mbar and amplitude of the oscillations of 100 mbar at a frequency of 68 Hz. After 15 min, the oscillations stopped and the catheter was deflated and moved to the other side, reinflated and treatment was continued for another 15 min.

### 2.3 HRV analysis

Continuous ECGs (Holter) were recorded using a Philips Zymed system (Philips Healthcare, Eindhoven, The Netherlands) with 200‐Hz sampling frequency and covered the time period from 30 min before meal intake to 3 hr after end of KOS treatment. Data analysis was performed by iCardiac Technologies, Rochester. Frequency‐domain HRV analysis was performed using the Fast Fourier Transformation of continuous NN series in windows of interest. Nonnormal beats were excluded and linear interpolation was applied. Normalized LF values were computed using standardized formula LFn = LF/(TP − VLF) and for HFn = HF/(TP − VLF), respectively.

After cleaning from ectopic beats, recordings were analyzed in 5‐min time windows for the following variables:

HR = heart rate (bpm)
NN = beat‐to‐beat interval of normal complexes (ms)
SDNN = standard deviation of NN intervals over a defined time period (ms)
RMSSD = the square root of the mean of the sum of the squares of the successive differences between adjacent NNs (ms)
LF = power in low‐frequency range (ms^2^; 0.04–0.15 Hz)
HF = power in high‐frequency range (ms^2^; 0.15–0.40 Hz)
VLF = power in very low‐frequency range (ms^2^; 0.0033–0.04 Hz)
LFn = power in low‐frequency range normalized
HFn = power in high‐frequency range normalized
LF/HF = ratio LF/HF

Five time windows of approximately 30‐ to 35‐min length, during which subjects were resting in a seated position, were selected for comparisons of HRV parameters (Figure 1):

- From start of the Holter recording to start of meal intake, representing fasting conditions;
- From start of meal intake to blood sampling immediately before KOS treatment, representing fed conditions;
- From start to end of KOS treatment;
- From 120 to 150 min after start of Holter recording, which meant that the period started 14–26 min after end of KOS treatment for different subjects;
- From 240 to 270 min after start of Holter recording, i.e., 2 hr after the start of period 4.

In each of the five periods, only data from 5‐min time windows that fell completely inside the period were included; as an example, results from 5‐min time windows in which the KOS treatment was either initiated or completed were not included in period III.

### 2.4 Statistical analysis of HRV variables

For each HRV variable, the individual mean value of the data for each period (I through V) was calculated and used for descriptive statistics and statistical comparisons. Descriptive statistics were used for both absolute and change‐from‐baseline summaries with period II used as baseline. For each HRV variable, a Friedman rank ANOVA was also performed to detect differences between the periods. A two‐sided *t*‐test was used to further analyze the change from baseline for each HRV variable statistically using an alpha of 10%, i.e., *p*‐values <.1 were viewed as statistically significant in a descriptive sense.

## 3 RESULTS

Twelve subjects completed all study periods on the treatment day. There were seven men and five women, with a mean age of 43 years (*SD*: 15.6; range 20–62). Ten subjects had no history of medical conditions, one subject had a benign psoriasis and one had undergone gastric bypass surgery. Baseline ECG was normal in all subjects except one, in whom a short PQ interval was noted. Two subjects had ongoing treatment with vitamin D and one female subject was on hormone replacement therapy; remaining subjects were not on any medical treatment.

**Figure 2:**
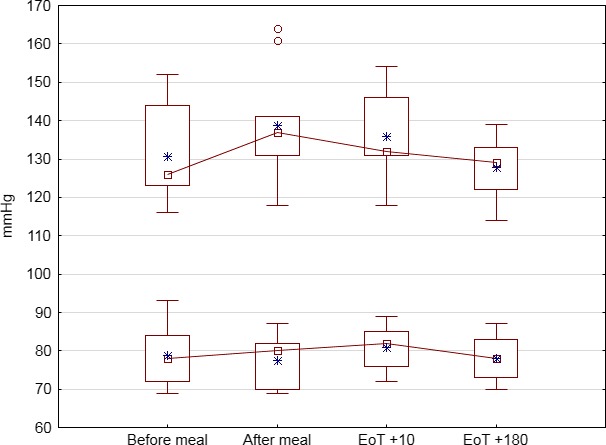
Blood pressure. Systolic and diastolic blood pressure at designated time points. After meal corresponded to immediately before kinetic oscillation stimulation (KOS) treatment. Box shows median, 25% and 75% quartiles, whiskers minimum and maximum values, and circles the arithmetic mean. EoT, end‐of‐treatment; EoT + 180, 180 min after EoT

Mean heart rate rose after meal intake, but remained stable during and after KOS treatment and blood pressure was also minimally affected by treatment. The mean changes from immediately before treatment to end of treatment were <1 bpm for pulse rate and <4 mmHg for systolic and diastolic blood pressure. Systolic and diastolic blood pressure across periods is shown in Figure 2. Respiratory rate showed minimal changes over the assessment period.

### 3.1 HRV variables

For the analysis of HRV parameters, the subject who had undergone gastric bypass was excluded, since this condition is known to have an effect on HRV (Maser, Lenhard, Peters, Irgau, & Wynn, 2013). A sensitivity analysis including this subject, however, demonstrated the same findings as described below. The number of available complete 5‐min time window recordings for the 11 subjects varied between 5 and 6 per subject for period I (mean 5.5) and period III (mean 5.7) and between 6 and for 7 per subject for period II (mean 6.4), period IV (mean 7.0), and period V (mean 6.9).

**Figure 3:**
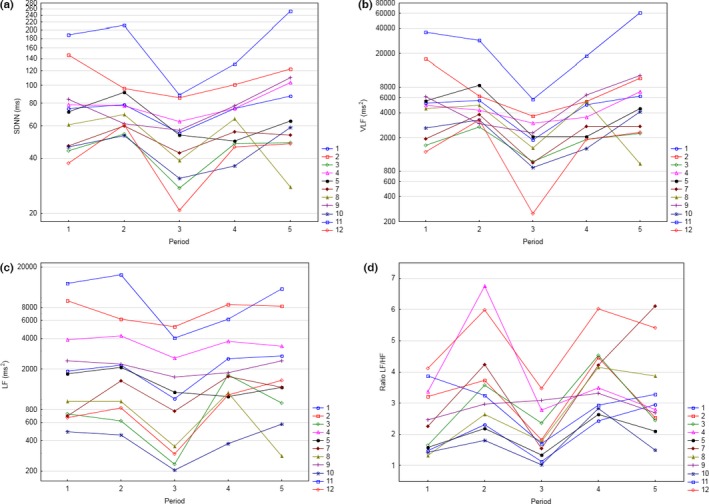
Heart rate variability. SDNN (a), VLF (b), LF (c) and LF/HF (d) for each subject across heart rate variability (HRV) periods. Period 2 was immediately before kinetic oscillation stimulation (KOS) treatment, whereas period 3 was during treatment

Descriptive statistics for time domain HRV variables is shown in Table 1 and for frequency‐domain variables in Table 2. Consistent with the vital signs assessment, heart rate increased during and after meal from a mean of approximately 61 bpm in the fasting state in period I to 68 bpm in period II, with corresponding changes of NN (corresponding to RR intervals, i.e., 60,000/HR; Table 1). The heart rate was essentially unchanged during and after KOS treatment, and returned toward baseline values in period V, 2–2.5 hr after KOS treatment with mean heart rate of 64 bpm. A clear and consistent reduction of SDNN was seen in all subjects during KOS treatment (Figure 3a) from a mean value immediately before treatment (period II) of 86 ± 41.5 ms (mean ± *SD*) to 55 ± 28.2 ms during treatment (period III), followed by a return toward pre‐KOS values in periods IV and V (Table 1). Using the period immediately before treatment (period II) as baseline, this corresponded to a statistically significant mean change‐from‐baseline (∆SDNN) of −31.2 ms (90% CI: −43.4 to −18.9; *p* = .001, Table 3). A reduction of the short‐term beat‐to‐beat difference measured as the RMSSD was also observed during KOS treatment with a reduction in mean values from 40 ± 25.0 to 33 ± 19.0 ms from Period II to III (Table 1), resulting in mean ∆RMSSD of −7 ms (90% CI: −13.7 to −0.2; *p* = .09, Table 3). In the frequency domain, a reduction in all parameters was also observed during KOS treatment (period III; Table 2). The reduction was most pronounced and consistently observed in all subjects for LF, VLF, and the ratio LF/HF (Figure 3b–d). The mean change‐from‐baseline from period II to 3 was −2,023 ms^2^ (90% CI: −4,027 to −19, *p* = .097) for LF (∆LF), −5,577 ms^2^ (90% CI: −8,939 to −2,215, *p* = .013) for ∆VLF, and −1.7 (90% CI: −2.3 to −1.1; *p* = .0004) for the ratio LF/HF (∆LF/HF; Table 3).

**Table 1:** HRV variables in the time domain by period

	Period	Mean	Median	*SD*	Range	CV%
HR (bpm)	I	61.07	65.22	8.07	51.19; 71.51	13.21
	II	68.26	69.85	7.13	56.88; 78.26	10.44
	III	67.82	67.29	9.47	53.35; 84.18	13.96
	IV	69.42	70.80	8.72	58.72; 82.55	12.56
	V	63.64	68.57	9.91	49.83; 77.04	15.57
RMSSD (ms)	I	44.81	39.66	26.61	20.89; 101.76	59.39
	II	40.14	38.10	25.83	19.51; 108.33	64.34
	III	33.18	30.90	18.98	11.52; 66.22	57.21
	IV	35.71	28.53	18.12	19.53; 67.88	50.75
	V	43.26	36.41	28.14	12.82; 103.04	65.04
SDNN (ms)	I	85.76	71.49	46.81	45.00; 200.25	54.58
	II	85.80	70.60	41.49	52.30; 201.63	48.36
	III	54.63	56.25	24.52	21.49; 107.20	44.89
	IV	73.89	68.67	27.10	46.47; 128.61	36.68
	V	90.13	82.40	57.51	32.14; 238.63	63.81

Based on descriptive statistics.

**Table 2:** HRV variables in the frequency domain by period

	Period	Mean	Median	*SD*	Range	CV%
HF (ms^2^)	I	1,178.52	856.54	1,234.99	198.94; 4,096.95	104.79
	II	1,135.97	755.21	1,555.59	160.84; 5,580.74	136.94
	III	935.08	716.70	940.29	91.10; 2,761.15	100.56
	IV	859.94	568.73	786.59	165.18; 2,556.69	91.47
	V	1,354.67	762.71	1,795.39	100.82; 5,938.36	132.53
LF (ms^2^)	I	3,502.95	1,532.06	4,917.37	625.67; 16,795.72	140.38
	II	3,659.61	2,093.02	4,837.74	466.47; 16,979.03	132.19
	III	1,636.64	1,174.56	1,586.81	253.96; 4,928.33	96.96
	IV	3,044.30	1,863.88	2,725.17	470.50; 8,963.81	89.52
	V	3,429.86	1,511.76	3,814.80	319.93; 12,466.81	111.22
VLF (ms^2^)	I	9,805.46	6,476.21	11,055.01	2,146.84; 39,641.44	112.74
	II	8,583.54	6,262.83	8,790.12	3,228.03; 34,392.30	102.41
	III	3,006.96	2,618.29	2,809.71	281.80; 10,876.48	93.44
	IV	5,851.56	5,179.07	4,437.76	1,955.02; 17,804.74	75.84
	V	11,228.60	6,363.23	15,971.53	1,352.31; 57,485.49	142.24
LF/HF	I	2.73	2.53	1.12	1.38; 4.59	40.92
	II	3.75	3.24	1.43	1.90; 6.29	38.23
	III	2.07	1.70	0.82	1.23; 3.82	39.41
	IV	4.06	3.99	1.39	2.10; 6.68	34.18
	V	3.32	2.98	1.42	1.60; 6.22	42.92

Based on descriptive statistics.

**Table 3:** Change‐from‐baseline (Period 2) of HRV parameters

HRV parameters	Period	Mean change	90% CI	*SD*	*p*‐Value*
∆HR bpm	III	−0.4	−2.19 to 1.31	3.20	.6577
	IV	1.2	−0.75 to 3.09	3.51	.2961
	V	−4.6	−7.46 to −1.77	5.21	.015
∆RMSSD, ms	III	−7.0	−13.73 to −0.19	12.39	.0918
	IV	−4.4	−11.56 to 2.69	13.03	.2852
	V	3.1	−2.34 to 8.57	9.98	.3247
∆SDNN, ms	III	−31.2	−43.42 to −18.92	22.42	.0010
	IV	−11.9	−24.69 to 0.88	23.40	.1223
	V	4.3	−8.95 to 17.61	24.30	.5679
∆HF, ms^2^	III	−201	−715.3 to 313.6	941.4	.4953
	IV	−276	−783.8 to 231.7	929.1	.3477
	V	219	−77.1 to 514.5	541.3	.2099
∆LF, ms^2^	III	−2,023	−4,027.3 to −18.7	3,667.7	.0973
	IV	−615	−2,301.4 to 1,070.8	3,085.5	.5233
	V	−230	−1,070.8 to 611.3	1,539.0	.6312
∆VLF, ms^2^	III	−55,767	−8,938.6 to −2,214.6	6,152.1	.0132
	IV	−2,733	−5,394.8 to −69.1	4,872.7	.0926
	V	2,645	−1,598.3 to 6,888.5	7,765.0	.2849
∆LF/HF	III	−1.7	−2.26 to −1.09	1.07	.0004
	IV	0.3	−0.29 to 0.91	1.10	.3740
	V	−0.4	−0.82 to −0.04	0.72	.0764

∆, Change‐from‐baseline (Period 2). *: Based on descriptive statistics. A *p*‐value <.10 was viewed as statistically significant.

The Friedman ANOVA (Table 4) confirmed that differences between periods were highly significant for all HRV except HF power. This significance is maintained even if multiplicity is taken into account. It can also be seen that when periods II (before KOS), III (during KOS), and IV (approximately 30 min after KOS) were analyzed separately, the heart rate change across these periods was not significant, whereas *p*‐values were <.05 for all HRV parameters except HF. A rank‐based ANOVA was also performed on data from the individual segments of each variable and demonstrated that the individual between period variability outweighs that within each period.

**Table 4:** Results of Friedman ANOVA

Variable	Periods II–V		Periods II–IV	
	χ^2^	*p*	χ^2^	*p*
HR	8.3	.039	0.5	.761
NN	8.1	.043	0.2	.913
SDNN	17.6	<.001	15.3	<.001
RMSSD	9.0	.029	6.7	.035
VLF	22.3	<.001	20.2	<.001
LF	17.7	<.001	16.5	<.001
HF	1.6	.664	0.2	.913
LF/HF	22.5	<.001	16.9	<.001

One of the subjects had a high incidence of ectopies with in total 386 supraventricular (SVES) and 125 ventricular extrasystoles during the 5‐hr recording. During the hour preceding KOS, there were 78 SVES, of which 58 were single ectopies, whereas only 15 SVES were recorded during the full hour which included KOS treatment. Another subject also had frequent SVES, but no such reduction was observed during treatment. In remaining 10 subjects, the frequency of ectopies was too low to allow a meaningful evaluation of their time course.

### 3.2 Biomarkers

**Figure 4:**
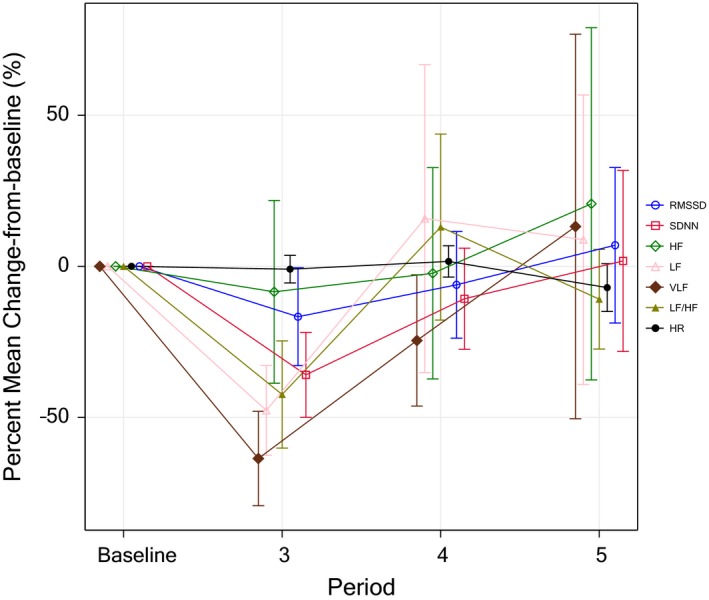
Change‐from‐baseline of heart rate and selected heart rate variability (HRV) parameters. Heart rate remained stable from baseline (period II) throughout observation periods during (period III), immediately after (period IV), and 3.5 hr after kinetic oscillation stimulation (KOS) treatment (period V). The reduction of HRV was pronounced during KOS treatment and rapidly returned toward baseline values thereafter. Values are mean ± standard deviation % change‐from‐baseline

Serum gastrin levels increased from a median value of 11.5 pmol/L in the fasted state to 53.5 pmol/L after meal intake. Immediately after KOS treatment, the median gastrin value was 24.5 pmol/L and 3 hr thereafter 15 pmol/L. Interleukin‐6 was the only cytokine demonstrating a clear change with a gradual increase from baseline up to 3 hr post end of treatment. Results for interleukin‐6 are shown in Figure 4.

### 3.3 Safety assessment

Kinetic oscillation stimulation treatment was well tolerated in all subjects without any treatment‐related adverse events. There were minimal changes over time in the spirometry variables vital capacity, forced expiratory volume over 1 s, forced vital capacity, and inspiratory capacity. There were no treatment‐related effects on hematology, coagulation, or clinical chemistry variables.

## 4 DISCUSSION

Kinetic oscillating stimulation has been shown to be effective in providing pain relief in patients with acute, ongoing migraine attacks (Juto & Hallin, 2015). The underlying mechanism for this treatment effect is largely unknown, but it has been hypothesized that it may be mediated through an alteration in autonomic balance (Juto & Hallin, 2015). The aim of this small and exploratory study was, therefore, to explore the effect of KOS treatment on markers of the autonomic nervous system, in addition to evaluating safety and tolerability, in healthy subjects. HRV, a widely recognized tool for the assessment of changes in autonomic balance, was, therefore, implemented during time windows of approximately 30 min prior to KOS treatment, during treatment, and immediately after and approximately 3.5 hr after treatment. The study demonstrated that KOS treatment had a pronounced effect on HRV with reductions of several parameters in both the time and frequency domain (Table 3). When comparing HRV parameters from the 30‐min time period immediately before KOS treatment (period II) with values during treatment (period III), there were statistically significant reductions of SDNN and RMSSD in the time domain and of LF, VLF, and the ratio LF/HF in the frequency domain (Table 3). These reductions were consistently seen in all subjects (Figure 3) and pronounced with mean decreases of 65% (median 58%) for VLF, 55% (median 43%) for LF, and 44% (median 48%) for LF/HF, and somewhat smaller reductions for SDNN and RMSDD with mean decrease of 36% and 18% (median 21% and 18%), respectively. The reduction of HRV was directly related to the KOS treatment with a rapid onset and resolution with return toward baseline values in periods IV and V (Figure 3). It is important to emphasize that the observed effects on HRV were seen in the absence of any significant heart rate changes; mean heart rate increased expectedly after the meal (Taubel, Wong, Naseem, Ferber, & Camm, 2012) and then remained stably around 68–69 bpm from the pretreatment (period II) through period IV, well after the 30‐min duration of the KOS treatment (Figure 4).

Treatment with KOS has been shown to be effective in subjects with nonallergic rhinitis and patients with migraine. In a placebo‐controlled study in 71 subjects with either nonallergic rhinitis or rhinitis medicamentosa, one treatment session with KOS reduced selected measures of nasal stuffiness during the week following the treatment (Juto & Axelsson, 2014). Whether this beneficial effect is the result of direct effects on the nasal mucosa or, as an example, results from improvement of local vascular function mediated through direct or indirect effects on the autonomic nervous system is not known. The authors pointed to the duration of the effect, lasting from days to weeks, and to reports indicating that patients with allergic rhinitis have a dysfunction of the autonomic nervous system (Ishman et al., 2007). In line with this, they suggested that potential mechanisms could involve a change in the balance between the sympathetic and parasympathetic parts of the autonomic nervous system (Bousquet et al., 2008; Ishman et al., 2007; Loehrl, 2007). A more recent, partially placebo‐controlled larger study in 207 patients with idiopathic rhinitis also demonstrated a beneficial effect by KOS but could not differentiate between treatment with high amplitude oscillations vs. low amplitude (Ehnhage et al., 2016). A beneficial effect has also been shown on pain intensity in patients with severe ongoing migraine treated in hospital (Juto & Hallin, 2015). Thirty‐five patients were randomized to KOS (*n* = 18) or placebo (*n* = 17; inserted balloon without pressure or oscillations) in a double‐blind design, and pain severity was assessed using symptom scores with a visual analog scale (0–10). Patients who received active treatment reported reduced pain with mean scores falling from 5.5 before KOS to 1.2 15 min after treatment; placebo subjects experienced a fall from 4.9 to 3.9, a difference between active and placebo of 3.3 points (95% CI: 2.3–4.4). The authors discuss that KOS may activate sensory nerve endings in the mucosa with afferents in the trigeminal nerve, and this may imply a role of the trigeminal parasympathetic autonomic reflex (May, 2005), which is involved in certain types of migraine (Avnon, Nitzan, Sprecher, Rogowski, & Yarnitsky, 2003; Goadsby & Lipton, 1997). Furthermore, the sphenopalatine ganglion is located a few millimeters underneath the nasal mucosa and may offer another route for sensory stimulation with connections to the hypothalamus and the autonomic nervous system (Khan, Schoenen, & Ashina, 2014; Schoenen et al., 2013).

It should be acknowledged that the mechanism leading to the beneficial effects seen in these relatively small studies with KOS is not well understood, but indirect effects on the autonomic nervous system may play a role (Li, Wang, Hallin, & Juto, 2016). It can be hypothesized that the pronounced reduction of HRV is either linked to an effect by KOS on breathing pattern or to a change in autonomic tone, through the so‐called trigemino‐cardiac reflex (Lemaitre, Chowdhury, & Schaller, 2015). The effect of spontaneous versus controlled breathing on HRV has been quite extensively studied (Malliani, Pagani, Lombardi, & Cerutti, 1991). In healthy subjects, breathing through the nostrils, both acutely and after training, lead to parasympathetic dominance with heart rate slowing and blood pressure effects (Jain, Srivastava, & Singhal, 2005; Srivastava, Jain, & Singhal, 2005). In a recent study, HRV was evaluated in 20 healthy subjects during spontaneous breathing (breathing rate 14.8 ± 0.7/min) and controlled breathing at the same (15 breaths/minute) and slower rate (6 breaths/minute) (Sasaki & Maruyama, 2014). Compared to spontaneous breathing, controlled breathing at the same rate resulted in an elevation of the heart rate and a reduction of the HF component of HRV, indicating a reduction of parasympathetic tone. During slow, controlled breathing, the heart rate was unchanged, whereas the LF/HF ratio increased substantially, possibly because some of the subcomponents of HF were synchronized with the breathing frequency and moved into the LF region. Similar findings of a pronounced increase of LF and thereby an increase of LF/HF have been reported in another study in 14 healthy subjects with spontaneous and controlled breathing in the supine position (Wang, Kuo, Li, Lai, & Yang, 2016). Based on this, and previous studies, it is clear that the breathing rate and probably pattern can impact HRV in a way that makes the interpretation of results difficult unless breathing is monitored or controlled. Even though our results do not match any of the described HRV patterns resulting from controlled breathing, we cannot exclude that changes on breathing rate or pattern caused by KOS contributed to the observed reduction of HRV in our study.

The nasal cavity is highly innervated including sensory, parasympathetic, and sympathetic nerves (Sahin‐Yilmaz & Naclerio, 2011; Widdicombe, 1986). Parasympathetic fibers originate in the superior salivary nucleus of the midbrain and then travel with the fibers of the seventh cranial nerve. Afferent signals from the nasal cavity may reach the central nervous system through several different pathways. The most apparent example of the close interconnection between afferent trigeminal nerve stimulation and cardiovascular response is the so‐called trigemino‐cardiac reflex, which in many parts resemble the diving reflex with bradycardia, reduction of limb blood flow and gradual rise of the arterial blood pressure (Chowdhury et al., 2015; Lemaitre et al., 2015). Less dramatic effects with a general decrease in HRV have been shown in healthy subjects during controlled breathing under different levels of nasal positive airway pressure (Valipour, Schneider, Kossler, Saliba, & Burghuber, 2005). The observed HRV reduction was observed across all frequency bands despite unaltered heart rate, which are findings that parallel those observed in this study. We, therefore, believe that one likely underlying mechanism explaining the observed HRV reduction is a change in the balance between sympathetic and parasympathetic autonomic tone.

In conclusion, treatment with KOS in this small, exploratory study in 12 healthy subjects led to substantial reduction of HRV, most pronounced for VLF, LF, and the ratio LF/HF, whereas heart rate remained unchanged. This reduction of HRV was consistently seen in all subjects, with rapid onset and return toward baseline values during posttreatment observation periods. It should be emphasized that the study was small and the findings warrant further research into the underlying mechanism.
